# Clinical Effectiveness of Herbal Oral Care Products in Periodontitis Patients: A Systematic Review

**DOI:** 10.3390/ijerph191610061

**Published:** 2022-08-15

**Authors:** Georgios S. Chatzopoulos, Panagiotis Karakostas, Stefania Kavakloglou, Andreana Assimopoulou, Panagiotis Barmpalexis, Lazaros Tsalikis

**Affiliations:** 1 Department of Preventive Dentistry, Periodontology and Implant Biology, School of Dentistry, Aristotle University of Thessaloniki, 54124 Thessaloniki, Greece; 2 Department of Developmental and Surgical Sciences, Division of Periodontology, School of Dentistry, University of Minnesota, Minneapolis, MN 55455, USA; 3 Faculty of Dentistry, Medical University of Sofia, 1431 Sofia, Bulgaria; 4 424 General Military Training Hospital, 56429 Thessaloniki, Greece; 5 School of Chemical Engineering, Aristotle University of Thessaloniki, 54636 Thessaloniki, Greece; 6 Department of Pharmaceutical Technology, School of Pharmacy, Aristotle University of Thessaloniki, 54636 Thessaloniki, Greece

**Keywords:** chlorhexidine, herbal, oral care, phytotherapy, scaling and root planing (SRP), systematic review

## Abstract

Background: The use of herbal products in oral cavity has shown an increased popularity and potential benefits due to their additional anti-inflammatory and antioxidant properties as well as the lack of side effects related to their use. Objective: To assess the clinical effectiveness of herbal dental products (mouthwash, dentifrice, gel) when compared to conventional products or placebo in periodontitis patients. Material and methods: A systematic review with 22 studies was carried out using MEDLINE/Pubmed, EMBASE and Web of Science databases in addition to hand searches. Randomized and non-randomized clinical trials that evaluated the effect of any herbal dental product and compared it with conventional products or placebo in periodontitis patients and published up to March 2022, were screened. Results: Herbal products used as adjuncts to scaling and root planing (SRP) or supragingival debridement (SPD) led to superior clinical outcomes than placebo or no adjuncts (8 studies). In conjunction with SRP, these products showed comparable outcomes with chlorhexidine (6 studies) or better (4 studies). When used as adjuncts to SPD, herbal oral care products demonstrated comparable outcomes with chlorhexidine and conventional products (4 studies). Conclusions: Within the limitations of this systematic review, herbal oral care products may play a key role in the management of periodontal disease. Further well-designed studies are needed to establish their efficacy.

## 1. Introduction

Periodontal disease is defined as a multifactorial inflammatory disease that is marked by destruction of the supporting tissues around teeth including periodontal ligament, cementum, alveolar bone, and it is the major cause of tooth loss, if left untreated [1,2]. Dental biofilm is the primary etiological factor of dental caries and periodontal diseases. The biofilm that is attached on the tooth surface consists of polymers and it is resistant to host defense and antibiotics. Effective biofilm destruction plays a key role in oral health. This can be performed by scaling and root planing (SRP) with or without the use of antibiotics and several agents and can be maintained by patient’s good oral hygiene [3].

According to the European Federation of Periodontology S3 level clinical practice guidelines, the elimination of the subgingival biofilm and calculus consists of subgingival debridement and the use of adjunctive agents (physical, chemical, host-modulating) and systemic or locally delivered antimicrobials [4,5,6,7,8]. Although non-surgical periodontal treatment is highly predictable and can improve the clinical periodontal status of periodontitis patients [9], 8–12% of patients within a population exhibit residual periodontal pockets that do not respond favorably to SRP alone [10]. This could be attributed to inadequate control of periodontal biofilm and poor oral hygiene [11]. To supplement the mechanical plaque control and augment daily oral hygiene, antimicrobial agents are included in toothpastes and mouthwashes to inhibit plaque accumulation and dental biofilm growth in areas that are not easily accessible with toothbrushing.

A number of antiseptics such as chlorhexidine (CHX) have been found to be effective against a wide range of Gram-positive and Gram-negative species as well as capable of penetrating the plaque biofilm [12]. CHX was originally discovered in the late 1940s by scientists looking for an antimalarial agent. It was further characterized as a broad-spectrum antimicrobial in the 1950s and its use in dentistry was popularized by a 1976 study showing long-term clinical benefits [13]. CHX has an antiplaque effect due to its binding to not only bacteria, but also salivary glycoproteins that interfere with pellicle formation and bacterial adsorption to teeth [14]. Due to its substantivity, it is able to prolong the duration of action and allow its slow release into the oral environment for up to 24 h [15]. CHX’s clinical efficacy as an antiplaque and anti-gingivitis agent has been confirmed by systematic reviews and meta-analyses [4,16,17]. However, CHX presents side effects such as the yellow-brown staining of the cervical third and interproximal areas, altered taste sensations, burning sensations, soreness and dryness of the oral soft tissues, desquamative lesions and ulcerations of the gingival mucosa, and increased supragingival calculus formation [18]. Due to these unfortunate side effects, alternative antiseptic agents are needed. 

Although conventional products and CHX control plaque and gingivitis, herbal-based products including dentifrices and mouthwashes have shown positive effects as anti-plaque and anti-gingivitis agents [19]. Due to the development of multidrug resistant pathogens and the necessity for economical, safe, and highly effective products resulted in the development of alternative oral care products derived from plants. Natural compounds have recently attracted a growing interest. There has been an increased public interest in natural or herbal health products, especially in patients with chronic diseases [20]. Products containing natural compounds have additional anti-inflammatory and antioxidant properties that could further benefit gingival health [21]. Natural products including *Acacia arabica*, *Aloe vera*, *Azadirachta indica*, *Curcuma longa*, *Cymbopogam*, *Camellia sinensis*, and *Ocimum sanctum* exhibit antimicrobial, anti-inflammatory, antiseptic and antifungal properties that could enhance wound healing [19]. However, the variety of formulations may hamper the effect of active and herbal agents. Individual herbs demonstrate moderate anti-septic action and therefore combining various herbs and chemicals may increase their anti-bacterial mechanisms [22]. Thus, they could be used to prevent and treat early stage periodontitis [22]. Different findings have been reported in the literature when chlorhexidine and herbal products are compared [23]. In a recent systematic review that included eight randomized clinical trials which compared the efficacy of herbal and CHX-based mouthrinses, three trials showed comparable results while one of them favoured CHX and another one favoured herbal mouthwashes [23]. Another aspect of this comparison should be the maintenance of the clinical outcome. The effectivenss of herbal oral care products were analysed in a meta-analysis which demonstrated that herbal products may have great short-term results, but non-herbal mouthwash may exhibit better long-term reduction of dental plaque [24]. This inconsistency may be a result of poorly designed studies that show increased heterogeneity due to the inclusion of populations with different characteristics as well as variable follow-up times [19]. 

Periodontitis is a chronic inflammatory disease [1,2] and patients diagnosed with periodontitis may benefit from the use of herbal-based oral products. Numerous natural products have been tested in the literature and to our knowledge no systematic review has focused on the use of these products in patients diagnosed with periodontitis. In addition, despite the increased number of trials testing the efficacy and safety of herbal dental products compared to conventional and CHX-based oral care products, the results are inconclusive and conflicting. Therefore, due to the increased popularity and the potential benefits of the herbal products, their efficacy should be systematically evaluated. We hypothesized that herbal oral care products demonstrate similar benefits with conventional dental products in patients diagnosed with periodontitis. The aim of this systematic review was to assess the clinical effectiveness of herbal dental products (mouthwash, dentifrice, gel) when compared to conventional products or placebo in periodontitis patients.

## 2. Materials and Methods

The present systematic review followed the PRISMA guidelines [25] and was registered in PROSPERO under the ID CRD42022316482.

### 2.1. PICO Question

A PICO (Population, Intervention, Comparison, and Outcome) question was utilized to formulate a focused question and guide the inclusion and exclusion criteria of the present systematic review. The study protocol and inclusion/exclusion criteria were specified in advance. The focused question is: “Are herbal oral products (mouthwash, dentifrice, gel) non-inferior/equally effective to conventional products in patients diagnosed with periodontitis?”

- Population: Adult human subjects diagnosed with periodontitis based on clinical examination (probing pocket depth and/or clinical attachment loss) and/or radiographic evaluation
- Intervention: Use of herbal dental products (mouthwash, dentifrice, gel) following SRP or supragingival debridement (SPD) or no additional periodontal treatment
- Comparison/Control: Use of non-herbal dental products (mouthwash, dentifrice, gel) or placebo following SRP or SPD or no additional periodontal treatment
- Outcome: Clinical periodontal parameters including probing pocket depth, gingival inflammation, dental plaque, clinical attachment loss.

### 2.2. Eligibility Criteria

All randomized and non-randomized clinical trials that evaluated the effect of any herbal dental products (mouthwash, dentifrice, gel) and compared it with conventional products including CHX or placebo in periodontitis patients were included in this systematic review. Included studies must have reported at least one clinical periodontal parameter including probing pocket depth (PPD), clinical attachment loss (CAL), gingival inflammation indices and dental plaque indices. The exclusion criteria were as follows: (1) publications in a language other than English, (2) case-series, case reports, letters to the Editor, commentaries and presentation abstracts, (3) individuals of <18 years, (4) studies on subgingival delivery of herbal gels, and (5) no full-text available. 

### 2.3. Search and Screening Strategy

Three electronic databases (MEDLINE-PubMed, EMBASE and Web of Science) were searched for articles relevant to the topic of this systematic review up to March 2022 by two independent evaluators (G.C., P.K.) in duplicate. In addition, manual searches of the reference lists of the included publications as well as online searches were performed. All search results were thoroughly screened and in duplicate for relevancy by the publications’ titles, abstracts and keywords. Irrelevant records were excluded. At the second stage of selection, all full-text articles identified during the first stage were evaluated for eligibility by two evaluators independently and in duplicate based on the inclusion and exclusion criteria. Disagreements regarding inclusion during the first and second stages of study selection were resolved by discussion. The level of agreement between the two examiners was calculated using kappa statistics. 

Combinations of controlled terms (MESH and EMTREE) and keywords were utilized: (herb OR herbal OR herbs OR natural OR organic) AND (Mouthwashes OR mouthwash OR oral rinse OR mouth rinse OR Toothpastes OR toothpaste OR tooth paste OR tooth pastes OR tooth powder OR tooth powders OR teeth powders OR teeth powder OR dentifrice OR dentifrices OR gel OR gels OR oral care product OR oral care products)) AND (Plaque OR dental plaque OR gingivitis OR gingival bleeding OR gingival index OR gingival inflammation OR bleeding on probing OR papillary bleeding OR bleeding index OR sulcus bleeding index OR plaque index OR interdental plaque OR inter proximal plaque OR dental deposit OR plaque deposit OR calculus OR biofilm OR periodontitis).

### 2.4. Data Extraction

Data extraction was carried out by two investigators (G.C., P.K) using templates to retrieve: (1) general information on the year of publication, country, study design; (2) information related to the treatment including type of periodontal therapy, adjunctive treatment groups, number of patients, periodontal parameters examined and follow-up period; (3) important clinical findings. 

### 2.5. Risk of Bias Assessment

The quality of the included randomized clinical trials was assessed using the revised tool for assessment of risk of bias in randomized trials (ROB 2.0 tool) [26]. In particular, the selected publications were assessed for the following parameters: bias arising from the randomization process, bias due to deviations from intended interventions, bias due to missing outcome data, bias in measurement of the outcome and bias in selection of the reported result [26]. The Methodological Index for Non-Randomized Studies (MINORS) tool was employed for non-randomized studies [27]. Each investigation could receive 0–2 points for each item with the total score ranging from 0–16. The scores can be classified as: 0–4, very low quality; 5–8, low quality; 9–12, moderate quality; and 13–16, high quality [28]. The risk of bias assessment was completed by two independent evaluators (G.C., P.K) and any discrepancy was resolved by discussion.

## 3. Results

**Figure 1:**
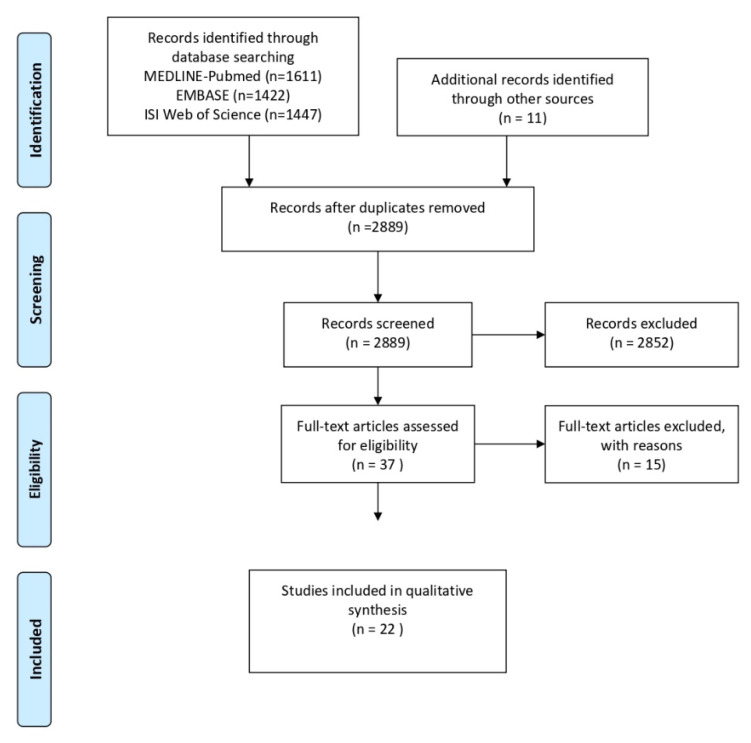
Preferred Reporting Items for Systematic Reviews and Meta-Analyses (PRISMA) flow diagram for study selection.

The Preferred Reporting Items for Systematic Reviews and Meta-Analyses (PRISMA) flow diagram for study selection is displayed in Figure 1. The initial electronic searches of the databases MEDLINE-Pubmed (n = 1611), EMBASE (n = 1422) and ISI Web of Science (n = 1447), as well as the additional records retried through other sources identified a total of 2889 studies after duplicates removal. Using titles and abstracts to screen the content, 2852 records were excluded, and 37 studies were assessed for eligibility. Fifteen studies were excluded based on the inclusion and exclusion criteria of this systematic review and 22 investigations were included in the qualitative synthesis. The Cohen’s kappa values for inter-reviewer agreement for the first and second rounds of study selection were 0.94 and 0.98, respectively. The reasons of exclusion were: (1) inclusion of human subjects with no diagnosis of periodontitis [29,30,31,32,33], (2) periodontal diagnosis based on partial clinical examination [21], (3) study protocol [34], (4) use of herbal gels subgingivally as local-drug delivery [35,36,37,38,39,40], (5) non-English publication [41] and (6) no full text available [42]. Meta-analysis was not possible due to the increased heterogeneity in the disease/health definitions, periodontal evaluation, follow-up times, study protocols, and the variety of herbs.

The characteristics of the included studies are shown in Table 1. All included studies were published between 2003 and 2022 [43,44,45,46,47,48,49,50,51,52,53,54,55,56,57,58,59,60,61,62,63,64] and the majority of them were randomized clinical trials [43,44,45,46,47,48,49,50,51,52,54,55,56,58,59,60,61,62,63,64] and conducted in India [43,45,46,47,48,51,52,53,55,63,64], while the rest of them were from Iran [44,49,59], Iraq [50,57], Pakistan [54], USA [56], Germany [58,60], Japan [61], and Italy [62]. The adjunctive therapies were herbal mouthwashes in 16 studies [43,44,45,49,50,52,53,54,55,56,57,59,60,62,63,64], solutions used with subgingival irrigators in 3 publications [46,47,51] and dentifrices in another three articles [48,58,61]. None of the included eligible investigations tested herbal gels. Various herbal products were tested in the included investigations including 1% *Matricaria chamomilla* [43], triphala (*Emblica officinalis*, *Terminalia chebula*, and *Terminalia belerica*) [45,52,53], *Saussurea lappa* extracts [46], 4% *Ocimums anctrum* [47], green tea [48,49,50], *Nigella sativa* [54], liquorice [57], *Stuckenia striata* [59], lemongrass oil [63], red ginseng [64], or combinations of different herbs [44,51,55,56,58,60,61,62].

SRP was completed in conjunction with the use of herbal products in 13 studies [43,44,45,46,47,48,49,50,52,53,54,55,57], whereas the rest of them performed SRP prior to the initiation of the study [51,56], or included SPD only [57,58,60,62,63,64], or no treatment [59,61]. All examined patients were diagnosed with chronic periodontitis. The severity and extend of the disease varied between the studies. Five studies included patients with “generalized chronic periodontitis” [43,45,53,55,64], while “chronic periodontitis” was reported in the inclusion criteria of eleven included investigations [44,46,47,51,52,54,56,57,59,60,61]. Other diagnoses included “mild to moderate periodontitis” [48], “generalized moderate chronic periodontitis” [49], “localised mild to moderate chronic periodontitis” [50], “slight and moderate chronic periodontitis” [58], “moderate or severe periodontitis” [62], “generalized severe periodontitis” [63]. The follow-up time ranged between 1 week and 6 months. Only one study reported data after six months [58]. Three-month results were shown in seven studies [43,51,52,56,60,62,63]. Various plaque indices and bleeding indices were used in the included studies. Plaque, bleeding/inflammation, and probing pocket depths were primarily reported. 

All included studies reported significant differences favoring the herbal products when compared to placebos or no adjuncts [43,44,46,50,53,62,63,64], apart from a comparative non-randomized clinical trial that reported no difference between SRP + liquorice mouthwash and SRP only [57]. When herbal oral care products were compared to CHX, comparable outcomes were found for 1% *Matricaria chamomilla* (MTC) [43], *Saussurea lappa* extracts [46], Triphala [53], polyherbal mouthwash HiOra^®^ [55], lemongrass [63] and red ginseng [64]. Comparable outcomes were shown between herbal and triclosan- and amine/stannous-based dentifrices [58]. Irrigation with 4% *Ocimum sanctum* or CHX led to similar reduction in gingival inflammation and plaque accumulation, whereas PPD and CAL exhibited superior improvement in the CHX group [47]. Subgingival irrigation with a polyherbal solution demonstrated significant plaque reduction, while CHX irrigation showed significant reduction in gingival inflammation [51].

Triphala and green tea mouthwashes also showed significantly better results than CHX [45,49,50,52] and conventional products [48]. A polyherbal mouthwash used for irrigation depicted significantly higher gingival inflammation reduction than conventional mouthwash used alone or as oral irrigation [60]. An herbal dentifrice also showed superior improvement than a conventional toothpaste [61]. CHX showed better clinical outcomes than herbal products in two investigations [44,56]. No differences between saline and *Nigella sativa* used as mouthwash were detected in a study [54].

**Figure 2:**
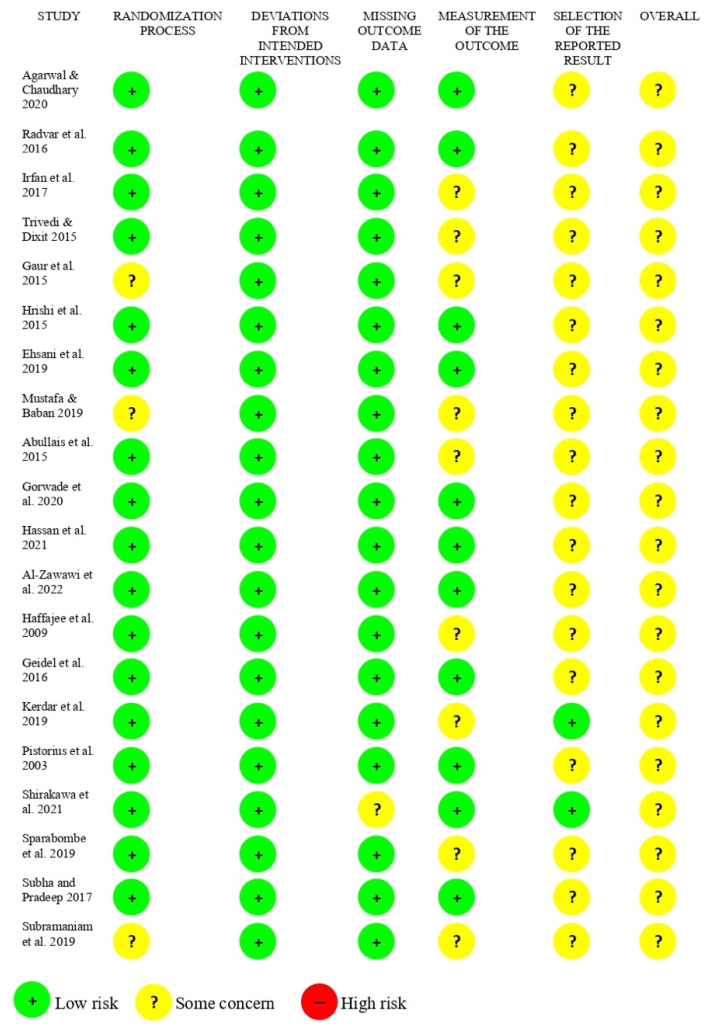
The ROB 2.0 tool was selected to evaluate the risk of bias of the included randomized clinical trials [41,42,43,44,45,46,47,48,49,50,52,53,54,56,57,58,59,60,61,62].

The risk of bias assessment of the included studies is presented in Figure 2 and Table 2. Twenty of the included studies were assessed according to the revised tool for assessment of bias in randomized clinical trials (RoB 2.0) [26]. Thirteen studies [43,44,48,49,52,54,55,58,59,60,61,63] were of low risk of bias in all but one domain, five studies [45,46,51,56,62] were of low risk of bias in all but two domains, while three studies [47,50,64] presented concerns in three domains. Overall, all studies presented concerns (Figure 2). The remaining two studies with non-randomized trials and were assessed for risk of bias using the MINORS tool (Table 2) [27]. One of these studies was rated with a score of 11 [53] indicating moderate quality, whereas the other one was rated with score of 8 [57] indicating low quality.

## 4. Discussion

Self-performed mechanical plaque removal has been shown to be insufficiently effective in adult patients and further measures are required to establish good oral hygiene [65]. Maintaining good plaque control using conventional products is difficult in the general population. In addition, presence of bleeding on probing plays a crucial role in attachment loss and progression of disease [66]. Therefore, it is important to use adjunctive antiseptic agents to improve oral hygiene and furthermore improve the periodontal treatment response. The use of adjunctive antiseptic agents is of paramount importance to supplement periodontal treatment, especially in situations that non-surgical periodontal treatment is not adequate [10,67,68]. CHX is considered the gold standard in periodontal antiseptic treatment, and it is widely used as an antimicrobial agent adjunct to SRP [69]. However, due to its unpleasant taste and undesirable tooth staining other products have been tested. Herbal products including mouthwash, dentifrice and gels have gained ground over the last years. It is believed that the active ingredients of herbal oral products can penetrate the biofilm and prevent plaque accumulation, therefore minimizing the colonization of bacterial on tooth surfaces [70]. Also, they exhibit antimicrobial efficacy against dental caries and periodontal pathogens, while lowering the development of drug resistance due to their synergistic combinations [71]. Herbal extracts may also inhibit osteoclast differentiation [72] as well as the expression of proinflammatory cytokines [73] and thus suppress bone resorption in periodontitis. The present study is the first systematic review to investigate the effect of herbal dental products compared to conventional products including CHX or placebo in periodontitis patients. 

This systematic review included 20 randomized clinical trials and 2 comparative non-randomized trials that examined the effect of herbal products as adjunctive treatment to SRP or SPD or no treatment. The use of these products was compared to CHX, conventional products or placebo. Among the herbs or plant extracts examined in the included studies, Triphala (3 studies) and green tea (3 studies) were the most studied. The majority of the included studies reported data with a relatively short follow-up time which was up to 6 weeks post-treatment. Only one study reported data after six months [58]. 

Based on the available literature, the main findings are:

- Herbal products (mouthwash and solution for irrigation) used in conjunction with SRP or SPD led to superior clinical outcomes when compared to placebo or no adjuncts (8 studies).
- Herbal oral care products (mouthwash, solution for irrigation and dentifrice) including 1% *Matricaria chamomilla*, *S. lappa* extracts, Triphala, 4% *Ocimum sanctum* and polyherbal mouthwashes/solutions used in conjunction with SRP shown comparable outcomes with CHX (6 studies) or significantly better (4 studies).
- Herbal dental products including lemongrass and red ginseng used as supplements of SPD resulted in comparable outcomes with CHX and conventional products (4 studies).
- None of the included studies investigated the effect of the use of supragingival application of herbal gels in periodontitis.
- Research regarding side effects is lacking.

To our knowledge, there are no previous systematic reviews evaluated the effect of herbal oral products including mouthwash, solution for oral irrigation and dentifrice in periodontitis patients. We aimed to include all types of study protocols including SRP, SPD and no treatment in order to accumulate the available evidence in a single publication. A recent study that examined the anti-inflammatory efficacy of curcumin as an adjunct to non-surgical periodontal treatment included 18 randomized clinical trials and demonstrated significant reduction in gingival index and sulcus bleeding index when compared to non-surgical treatment alone [74]. When curcumin was compared to CHX as adjunct to SRP, it showed similar reductions of PPD, CAL, gingival index and plaque index with CHX [75]. 

In patients undergoing fixed orthodontic treatment, a systematic review by Kommuri et al., assessed the clinical periodontal parameters after the use of CHX and herbal mouthwashes [23]. Due to the limited number of eligible studies and the conflicting results, the research question remained inconclusive with the majority of the included studies showing comparable effects between herbal mouthwashes and CHX. In another systematic review that included subjects with gingivitis only, herbal mouthwashes used as an adjunct to oral hygiene led to significantly improved outcome regarding dental plaque and gingival inflammation reduction when compared to placebo [76]. The effect of herbal products should be interpreted with caution due to the inclusion of studies with short to intermediate follow-up time (fourteen days to three months) [76]. When the effect of herbal dentifrice was compared to conventional products, a systematic review concluded that there were no differences in plaque and gingival inflammation short- and long-term [77]. However, it is important to highlight the increased heterogeneity and the high risk of bias of the included studies [77]. 

Superior results were found in another systematic review that assessed the effectiveness of herbal oral care products in the reduction of dental plaque and gingivitis [24]. Herbal toothpastes and mouth rinses demonstrated significantly higher plaque reduction compared to non-herbal ones. Herbs including *Camelia sinensis*, *Azadirachta indica*, *Anacardium occidentale* Linn, *Schinus terebinthifolius* and *Curcuma longa* resulted in higher plaque and inflammation reduction than CHX. Natural plant-based antimicrobials including *Vitis vinifera*, Pinus species, *Coffea canephora*, *Camellia sinensis*, *Vaccinium macrocarpon*, *Galla chinensis*, *Caesalpinia ferrea Martius*, *Psidium cattleianum*, representative Brazilian plants and manuka honey exhibit antimicrobial effects eliminating multispecies oral biofilms and they can be used in the treatment of dental diseases effectively [78]. Based on the findings of the present systematic review, herbal oral care products may be used in conjunction with SRP and SPD in daily practice to enhance the clinical treatment outcome. This is a result of the strong antimicrobial activity of these herbal products and their synergistic effect again oral biofilm. Herbs may also inhibit the growth of periodontal pathogens, reduce the inflammatory response of the host to the bacteria and finally inhibit innate and adaptive immune responses in periodontal tissues [74]. For example, the use of Salvadora persica extract has significant anti-streptococcal and anti-lactobacilli effects and is associated with a significant reduction in plaque score and cariogenic bacterial count [79]. Similarly, liquid neem extract significantly reduced the Lactobacillus and S. mutans counts in gingivitis patients that used neem gel [80].

The subgingival delivery of natural products after non-surgical periodontal therapy has also been utilized to treat periodontal diseases. Naturally-occurring agents have been used to control the microbial challenge and host response preventing bone destruction [81]. Natural products used subgingivally are effective in PPD reduction and CAL gain as well as in eliminating the periodontal inflammation significantly compared to scaling and root planing alone or with placebo [82]. Novel herbal-based gels such as 15% natural chitosan can be used in the management of intrabony defects as an effective product for periodontal regeneration [83]. Therefore, the local use of herb-based products could also promote additional benefits in periodontal treatment [84]. This topic was not in the scope of the present systematic review and therefore studies on local-drug delivery of herbal gels applied subgingivally were excluded.

Important limitations of the present systematic review were the variability among the studies and the heterogeneity in various parameters including study protocols and interventions, disease severity and extent at baseline (periodontitis diagnosis), natural product action, treatment regimen, and follow-up duration. In the majority of the included studies, the treatment with natural products (mouthwash, dentifrice or subgingival irrigation with herbal based solution) was carried out during the initial active non-surgical periodontal treatment. Some clinical studies applied the natural products during the supportive periodontal therapy and were used in conjunction with SPD. Herbal products were also used without any type of periodontal therapy in two studies [59,61]. However, this systematic review reports the current knowledge regarding the use of different herbal products in periodontitis patients. Future studies should follow a standardized protocol of assessment of clinical effectiveness regarding plaque accumulation, bleeding/gingival inflammation and periodontal conditions, similar methodology and follow-up period. Uniform data reporting is key for study comparisons. Further research should compare the treatment outcomes when different adjunctive herbal oral products are used in different periodontitis stages. It is crucial to develop guidelines, supported by evidence, for the use of herbal products in dentistry.

## 5. Conclusions

In conclusion, within the limitation of this systematic review, the use of herbal oral care products in conjunction with SRP or SPD may promote additional benefits when compared to periodontal treatment alone. In addition, there is evidence which supports that herbal products have comparable clinical outcomes with CHX and conventional products with no relevant adverse effects. The use of adjunctive plant-derived actives could substitute chemicals in the management of periodontitis patients due to their significant benefits in clinical outcomes and the absence of relevant side effects and can be recommended as an alternative. Future well-designed and powered investigations should follow standardized protocols to identify adequately the effect of different herbal dental products.

## Figures and Tables

**Table 1:** The characteristics of the included studies.

Study	Year of Publication	Country	Study Design	Adjunctive Therapy	Groups	Number of Subjects (Males/Females)	Periodontal Diagnosis	Parameters	Follow-Up	Conclusions
Agarwal & Chaudhary [43]	2020	India	RCT	Mouthwash	A. SRP + 1% *Matricaria chamomilla* (MTC) B. SRP + 0.12% CHX C. SRP + placebo	A. 25 (12/13) B. 25 (12/13) C. 25 (15/10)	Generalized chronic periodontitis	PI, GI, sulcus bleeding, PPD, CAL, GRE, SI	6 weeks, 3 months	MTC significant benefits over placebo and comparable to CHX.
Radvar et al. [44]	2016	Iran	RCT	Mouthwash	A.SRP + herbal (*Althaea officinalis, Salix alba* and *Malva* *Silvestris* extracts) B. SRP + CHX C. SRP + placebo	30	Chronic periodontitis	PPD, BOP, CAL	6 weeks	Herbal mouthwash significant benefits over placebo. CHX better than herbal.
Irfan et al. [45]	2017	India	RCT	Mouthwash	A. SRP + triphala B. SRP + 0.2% CHX	A. 25 B. 25	Generalized chronic periodontitis	PI, GI	7, 30, 45 days	Triphala mouthwash effective in reducing plaque and gingival inflammation. Triphala more effective in reducing inflammation.
Trivedi & Dixit [46]	2015	India	RCT	Subgingival irrigation with solution	A. SRP + *S. lappa* extracts B. SRP + 0.2% CHX C. SRP alone	30 Periodontal pockets: A. 180 B. 180 C. 180	Chronic periodontitis	Modified SBI, PPD, CAL	7, 14, 28, 42 days	Irrigation with *S. lappa* significant benefits over SRP alone. *S. lappa* and CHX similar results. *S. lappa* better in 4–5 mm pockets.
Gaur et al. [47]	2015	India	RCT	Subgingival irrigation with solution	A. SRP + 4% *Ocimum sanctum* B. SRP + 0.2% CHX	30 Periodontal pockets: A. 15 B. 15	Chronic periodontitis	PI, GI, PPD, CAL	30 days	Irrigation with *Ocimum sanctum* showed similar significant benefits in gingival inflammation and plaque with CHX. CHX was superior in PPD and CAL reduction.
Hrishi et al. [48]	2015	India	RCT	Dentifrice	A. SRP + green tea B. SRP + triclosan-containing	30 (13/17) A. 15 B. 15	Mild to moderate periodontitis	PI, GI, PPD, BOP, CAL	4 weeks	Green tea showed greater reduction of gingival inflammation and CAL.
Ehsani et al. [49]	2019	Iran	RCT	Mouthwash	A. SRP + 0.05% green tea B. SRP + 0.2% CHX	40 (19/21) A. 20 (4/16) B. 20 (15/5)	Generalized moderate chronic periodontitis	BOP, PI, GI, PPD, CAL	7 and 21 days	Green tea was more effective to CHX at 3 weeks.
Mustafa &Baban [50]	2019	Iraq	RCT	Mouthwash	A. SRP + 5% green tea B. SRP + 0.12% CHX C. SRP only	45 (27/18) A. 15 B. 15 C. 15	Localized mild to moderate chronic periodontitis	GI, PI, GBI, PPD, CAL	30 days	Green tea significantly improved all periodontal parameters compared to CHX or SRP only.
Abullais et al. [51]	2015	India	RCT	Subgingival irrigation with solution	A. *Punica granatum* Linn., *Piper nigrum* Linn., detoxified copper sulfate B. 0.2% CHX (SRP completed 1 month earlier)	30 A. 15 B. 15	Chronic periodontitis (residual pockets following SRP)	PI, SBI, PPD	15, 30, 60, 90 days	Irrigation with herbal solution resulted in significant plaque reduction. CHX led to significant reduction in gingival inflammation.
Gorwade et al. [52]	2020	India	RCT	Mouthwash	A. SRP + Triphala B. SRP+ Bakul C. SRP + CHX	90 A. 30 B. 30 C. 30	Chronic periodontitis	GI, OHI, PI, PPD, CAL, papillary bleeding index	2, 4, 6, 8, 12 weeks	Triphala resulted in significantly greater plaque reduction at 3 months thanBakul and CHX.
Desai & Debnath [53]	2010	India	Non-RCT	Mouthwash	A. SRP + Triphala B. SRP + CHX C. SRP only	24 A. 8 B. 8 C. 8	Generalized chronic periodontitis	PI, GI, OHI, Periodontal index	7, 30, 45 days	Triphala showed significant reduction in periodontal indices compared to SRP alone. Similar outcome between Triphala and CHX.
Hassan et al. [54]	2021	Pakistan	RCT	Mouthwash	A. SRP + *Nigella sativa* B. SRP + saline	50 A. 25 B. 25	Chronic periodontitis	PPD, CAL, PI, BOP	2 weeks	Both *Nigella sativa* and saline had a significant beneficial effect. No difference between the mouthwashes.
Al-Zawawi et al. [55]	2022	India	RCT	Mouthwash	A. SRP + Herbal (Himalaya Drug Company, HiOra^®^) B. SRP + 2% saline C. SRP + 0.12% CHX	37 (27/10) A. 12 (9/3) B. 13 (8/5) C. 12 (10/2)	Generalized chronic periodontitis	PI, GI, PPD, CAL	6 weeks	Significant improvement in PI, GI and PPD at 6 weeks. No differences between the mouthwashes.
Haffajee et al. [56]	2009	USA	RCT	Mouthwash	A. Listerine Cool Mint (essential oil) B. Peridex (CHX) C. The Natural Dentist Healthy Gums Oral rinse (herbal) D. The Natural Dentist Healthy Gums minus bloodroot (herbal)	122 A. 28 B. 31 C. 29 D. 28	Chronic periodontitis (residual pockets following SRP)	GI, PI, BOP, PD, CAL	3 months	Both herbal mouthwashes reduced plaque significantly. No significant change in gingival inflammation. CHX significantly better results.
Ali & Mohammed [57]	2016	Iraq	Non-RCT	Mouthwash	A.SRP + liquorice mouthwash B.SRP	A. 15 B. 15	Chronic periodontitis	PI, GI	1 week	Decrease of plaque & gingival inflammation. No significant differences between SRP and SRP + liquorice.
Geidel et al. [58]	2015	Germany	RCT	Dentifrice	A. SPD + Herbal toothpaste B. SPD + triclosan/copolymer toothpaste C. SPD + amine/stannous fluoride toothpaste	A. 25 B. 26 C. 25	Slight & moderate chronic periodontitis	OHI, API, SBI, BOP, PPD, CAL	6, 12, 24 weeks	The herbal toothpaste as good as the control toothpastes.
Kerdar et al. [59]	2019	Iran	RCT	Mouthwash	A. Herbal (Hydro alcoholic extract of *Stuckenia striata*) mouthwash B.Irsha mouthwash No periodontal treatment	A. 25 B. 25	Chronic periodontitis	PI, PD, BOP	2, 4 weeks	Herbal mouthwash effective to chronic periodontitis and more potent compared to Irsha mouthwash.
Pistorius et al. [60]	2003	Germany	RCT	Mouthwash	A. SPD + oral irrigator + herbal mouthwash B. SPD + oral irrigator + conventional mouthwash C. SPD + conventional mouthwash	A. 34 B. 29 C. 26	Chronic periodontitis	GI, SBI, PI, PD	4, 8, 12 weeks	Herbal mouthwash group showed significantly higher SBI & GI reduction.
Shirakawa et al. [61]	2021	Japan	RCT	Dentifrice	A. Herbal toothpaste B. Control toothpaste No periodontal treatment	A. 37 B. 37	Chronic periodontitis	GI, PD, BOP, Plaque control record	2, 4 weeks	Significant improvement of clinical parameters when herbal toothpaste used.
Sparabombe et al. [62]	2019	Italy	RCT	Mouthwash	A. SPD + herbal mouthwash B. SPD + placebo mouthwash	A. 20 B. 20	Moderate or severe periodontitis	FMBS, FMPS, PD and CAL	12 weeks	Herbal mouthwash led tosignificantly higher reduction of bleeding score and plaque accumulation.
Subha and Pradeep [63]	2017	India	RCT	Mouthwash	A. SPD+ lemongrass oil mouthwash B. SPD + CHX mouthwash C. SPD only	A. 15 B. 15 C. 15	Generalized severe periodontitis	PPD, CAL, c-reactive protein, total cholesterol, high and low density lipid, triglycerides	12 weeks	Lemongrass oil mouthwash can be a good alternative in chronic periodontitis. Significantly higher PPD, CAL reduction in Herbal and CHX groups than SPD only.
Subramaniam et al. [64]	2019	India	RCT	Mouthwash	A. SPD + Red Ginseng mouthwash B. SPD + CHX mouthwash C. SPD+ placebo mouthwash	A. 10 B. 10 C. 10	Generalized chronic periodontitis	GI, FMBS, FMPS, PPD, CAL	4 weeks	Red Ginseng mouthwash has comparable effects to CHXand it is significantly better in GI and FMBS reduction than placebo.

Haffajee et al.: Natural Dentist Healthy Gums Oral Rinse contain several naturally occurring anti-inflammatory agents, such as aloe vera and calendula, and antimicrobial agents such as goldenseal and grapefruit seed. Geidel et al.: Herbal dentifrice containing a mixture of fermented herbs and natural essential oils, *Melia azadiracht* extract, *Krameria triandra* extract, propolis cera extract, *Ricinus communis* oil, *Salvia officinalis* extract, *Chamomilla recutita* extract, *Stevia rebaudiana*, *Aloe barbadensis* gel, *Commiphora myrrha* extract. Pistorius et al.: Herbal mouthrinse containing Salvia officinalis, Methapiperita, menthol, Matricaria chamomilla, Commiphora myrrha, Carvumcarvi, Eugenia caryoophyllus, and Echinacea purpurea. Shirakawa et al.: Herbal dentifrice containing rhatany tincture (1.25%), chamomile tincture (1.25%), and myrrh tincture (0.62%). Sparabombe et al.: The polyherbal mouthwash containing Propolis resin extract (1:3), *Plantago lanceolata* leaves extract (1:10), *Salvia officinalis* leaves extract (1:1) and 1.75% of essential oils from *Salvia officinalis*, *Syzygiumaromaticum* buds, *Mentha piperita* leaves, *Commiphora myrrha* oleoresin and *Pistacia lentiscus* oleoresin. Abbreviations: API: Approximal plaque index. BOP: Bleeding on probing. CAL: Clinical attachment loss. CHX: Chlorhexidine. FMBS: Full-mouth bleeding score FMPS: Full-mouth plaque score GI: Gingival index MTC: Matricaria chamomilla OHI: Oral hygiene index PI: Plaque index PPD: Probing pocket depth RCT: Randomized clinical trial SBI: Sulcus bleeding index SPD: Supragingival debridement SRP: Scaling and root planing.

**Table 2:** Risk of bias assessment of non-randomized clinical trials using the Methodological Index for Non-Randomized Studies (MINORS) tool.

	Desai & Debnath [53]	Ali & Mohammed [57]
Clearly stated aim	2	2
Inclusion of consecutive patients	1	1
Prospective data collection	1	1
Endpoints appropriate to study aim	2	1
Unbiased assessment of study endpoints	1	1
Follow-up period appropriate	2	0
<5% lost to follow-up	2	2
Prospective calculation of study size	0	0
Total	11	8

## Data Availability

All data generated or analysed during this study are included in the published review article.
